# Clinical Management in NSCLC Patients With EGFR Mutation After Osimertinib Progression With Unknown Resistance Mechanisms

**DOI:** 10.1111/crj.70025

**Published:** 2024-10-15

**Authors:** Xin Liao, Tingting He, Xiong Wan, Pian Liu, Jing Li, Yong He, Yubo Wang

**Affiliations:** ^1^ Department of Respiratory and Critical Care Medicine Chongqing University Jiangjin Hospital Chongqing China; ^2^ Department of Respiratory and Critical Care Medicine Daping Hospital, Army Medical University Chongqing China

**Keywords:** chemo‐immunotherapy, EGFR mutation, NSCLC, osimertinib, resistance

## Abstract

**Background:**

Osimertinib is approved as a standard treatment for non‐small cell lung cancer (NSCLC) patients with epidermal growth factor receptor (EGFR) mutation by FDA. However, the mechanisms of resistance for nearly half of patients after osimertinib progression are still unknown, and the optimal therapies for these patients are still controversial. In this retrospective study, we compared efficacy and safety between immunotherapy + chemotherapy, chemotherapy alone, and osimertinib + bevacizumab in NSCLC patients after osimertinib progression with unknown resistance mechanisms.

**Methods:**

Advanced NSCLC patients with unknown resistance mechanisms after osimertinib progression were retrospectively reviewed and divided into immunotherapy + chemotherapy, chemotherapy alone, and osimertinib + bevacizumab treatment groups according to the treatment they received after osimertinib progression. Clinicopathological features, objective response rate (ORR), progression‐free survival (PFS), and overall survival (OS) were compared between groups.

**Results:**

A total of 121 patients were enrolled in this study, 22 in the immunotherapy + chemotherapy group, 72 in the chemotherapy group, and 27 in the osimertinib + bevacizumab group. The ORR was much higher in the immunotherapy + chemotherapy group compared with chemotherapy or osimertinib + bevacizumab group (55.56% vs. 14.81% vs. 0% in patients after progression on 1st line osimertinib treatment; 30.77% vs. 6.67% vs. 13.33% in patients after progression on 2nd/3rd line osimertinib treatment). Median PFS was also significantly longer in the immunotherapy + chemotherapy group compared with other groups (8.2 months vs. 4.0 months vs. 6.0 months in all patients, *p* = 0.0066). The median OS did not reach remarkable difference among groups, although osimertinib + bevacizumab group had a numerically longer median OS (37.0 months vs. 37.0 months vs. 47.6 months in all patients, *p* = 0.6357). Compared with immunotherapy + chemotherapy and chemotherapy, treatment‐related adverse events (AEs) of osimertinib + bevacizumab were milder, especially in AEs related to gastrointestinal and bone marrow suppression.

**Conclusion:**

Our study provides clinical evidence that NSCLC patients after osimertinib progression with unknown resistance mechanisms may benefit from immunotherapy + chemotherapy, with higher ORR and longer PFS compared with osimertinib + bevacizumab or chemotherapy groups. Osimertinib + bevacizumab treatment was also an optional option for patients because OS was numerically longer and safer in this group.

## Graphical abstract

Immunotherapy + chemotherapy may be a better choice for patients with non‐small cell lung cancer whose resistance mechanism is unknown after osimertinib resistance.

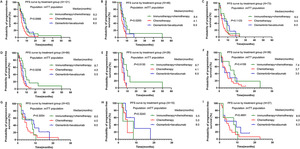

## 1 Introduction

Non‐small‐cell lung cancer (NSCLC), which accounts for about 85% of all lung cancers, is one of the most commonly diagnosed cancers with high mortality worldwide [1]. Epidermal growth factor receptor (EGFR) mutations are commonly detected in NSCLC, with a higher rate in Asian patients (about 50%) than Caucasian patients (10%–15%) [2]. The discovery of EGFR tyrosine kinase inhibitor (EGFR‐TKI) is an important milestone in the development of tumor‐targeted therapy in NSCLC and greatly improves the outcomes of NSCLC patients. EGFR exon 19 deletion (19DEL) mutation and exon 21 L858R mutation, also known as “common mutation”, account for about 90% of all EGFR mutations [3]. Numerous studies have shown significant efficacy of first‐ and second‐generation EGFR‐TKIs (gefitinib, erlotinib, icotinib, afatinib, dacomitinib) in patients with EGFR common mutations [4, 5, 6, 7, 8]. However, relapse often occurs within 1 year, with EGFR T790M mutation being the most commonly acquired resistance mechanism in patients treated with first‐ or second‐generation EGFR‐TKIs [9, 10].

Osimertinib, the first third‐generation EGFR‐TKI, can overcome EGFR T790M mutation after resistance to first‐ or second‐generation EGFR‐TKIs [11, 12]. In the randomized double‐blind Phase 3 FLAURA study, osimertinib showed prolonged progression‐free survival (PFS) and overall survival (OS) compared with those treated with gefitinib as first‐line treatment (median PFS: 18.9 months vs. 10.2 months, median OS: 38.6 months vs. 31.8 months) [13, 14]. Based on these results, osimertinib is approved as a first‐line or later‐line treatment for NSCLC patients with EGFR common mutations. Unfortunately, resistance to osimertinib inevitably developed in these patients.

Mechanisms of resistance to osimertinib are usually divided into EGFR‐dependent mechanisms (EGFR amplification, EGFR C797S mutation, etc.) and EGFR‐independent mechanisms (c‐MET amplification, BRAF mutation, histological transformation, etc.) [15, 16]. However, the resistance mechanisms for nearly half of patients after osimertinib progression are still unknown, and chemotherapy is often used to overcome unknown resistance mechanisms with no chance of using precise targeted therapy in the later stage [17]. Therefore, it is important to explore a better treatment strategy for patients after osimertinib progression with unknown resistance mechanisms. In recent years, immune checkpoint inhibitor (ICI) targeting programmed death‐1 (PD‐1) and PD‐1 ligand (PD‐L1) has transformed standard treatment for NSCLC patients without driver mutations [18, 19, 20]. Patients with EGFR mutation hardly benefit from monotherapy with ICI, but the situation can be improved when ICI is combined with other therapies. The IMPOWER 150 study demonstrated clinical benefit of a PD‐L1 inhibitor plus chemotherapy plus antiangiogenic therapy in patients after progression on EGFR‐TKIs, but no data are available for patients after progression on osimertinib [21]. ORIENT‐31 showed the benefit of sintilimab plus bevacizumab biosimilar plus chemotherapy in patients with EGFR‐mutated NSCLC who progressed on treatment with tyrosine‐kinase inhibitors [22]. Moreover, previous study indicated that osimertinib plus bevacizumab can also resolve resistance to some extent in patients after progression on osimertinib [23]. However, few researchers have compared the efficacy and safety of different therapies including immunotherapy plus chemotherapy, chemotherapy, osimertinib plus bevacizumab in patients following osimertinib progression with unknown resistance mechanims. Here, we aimed to compare the efficacy and safety of these three treatments in patients with EGFR‐mutant NSCLC after osimertinib progression with an unknown resistance mechanism.

## 2 Methods

### 2.1 Patients and Data Collection

In this retrospective study, patients pathologically diagnosed with advanced NSCLC harboring EGFR mutation after progression on osimertinib from 1st line or 2nd/3rd line treatment at Jiangjin Hospital and Daping Hospital from 2016 to 2022 were enrolled. Patients were eligible for inclusion if they progressed on osimertinib with unknown resistance mechanism after next generation sequencing (NGS) testing. Following osimertinib treatment, patients should immediately receive immunotherapy plus chemotherapy or chemotherapy or osimertinib plus bevacizumab as next line treatment. Patients with on‐target or off‐target resistance mechanism of osimertinib such as EGFR C797S mutation, MET amplification, ALK fusion, BRAF V600E mutation, and so on were excluded from the study. Patients who went on to switch to another targeted therapy following osimertinib immediately were also excluded. Patients with osimertinib plus (chemotherapy, radiotherapy, or other TKIs) and patients with anlotinib were also excluded. The patients in 2nd/3rd line osimertinib treatment group might receive chemotherapy or first‐generation or second‐generation EGFR‐TKIs in front line treatment. A total of 121 patients were enrolled for follow‐up until July 2023 or death.

Demographic data and clinical characteristics, including age, sex, smoking status, pathological type, stage, central nervous metastasis, and baseline EGFR mutation status, were collected from electronic medical records. Depending on the treatment after progression on osimertinib, patients were divided into three groups: (1) immunotherapy + chemotherapy group (*n* = 22): patients received tislelizumab (200 mg every 3 weeks) or pembrolizumab (200 mg every 3 weeks), cisplatin at a dose of 75 mg/m^2^ combined with either pemetrexed (500 mg/m^2^) or paclitaxel (175 mg/m^2^) once in a 28‐day cycle; (2) chemotherapy group (*n* = 72): patients received cisplatin (75 mg/m^2^) combined with either pemetrexed (500 mg/m^2^) or paclitaxel (175 mg/m^2^); (3) osimertinib + bevacizumab group (*n* = 27): patients received osimertinib 80 mg qd and bevacizumab 7.5 mg/kg every 3 weeks. Tumor response was evaluated according to Response Evaluation Criteria in Solid Tumors 1.1 criteria every 2–3 months. PFS was defined as the time from initiation of therapy to disease progression or death. OS was defined as the time from the beginning of treatment to the date of death or last follow‐up. Treatment‐related adverse events (TRAEs) were graded according to the Common Terminology Criteria for Adverse Events (CTCAE) version 5.

### 2.2 Statistical Analysis

Differences in clinicopathological characteristics between different groups were assessed by chi‐square testing. PFS and OS were estimated using Kaplan–Meier method and compared using the log‐rank test. All statistical analyses were performed by IBM SPSS Statistics for Windows (version 23.0). All reported *p*‐values were two‐tailed, and the difference was considered statistically significant at *p* < 0.05.

## 3 Results

### 3.1 Patients' Characteristics

Patients in different treatment groups after osimertinib progression were further divided into two subgroups according to osimertinib treatment lines (1st line or 2nd/3rd line). Clinicopathological characteristics were compared among these groups.

All baseline characteristics except smoking history in the three groups were well balanced (all *p* > 0.05). In the immunotherapy + chemotherapy group, there were more patients with smoking history (63.6%) than in the chemotherapy group (44.4%) and osimertinib + bevacizumab group (22.2%) (*p* = 0.013). The majority of patients were female (59.1% vs. 66.7% vs. 63.0%, *p* = 0.796), adenocarcinoma (100% vs. 98.6% vs. 96.3%, *p* = 0.648), stage IV (100% vs. 95.8% vs. 88.9%, *p* = 0.236), and EGFR 19DEL mutation (63.6% vs. 52.8% vs. 51.9%, *p* = 0.642) in the three groups. Central nervous metastasis rates were similar among the groups (18.2% vs. 16.7% vs. 11.1%, *p* = 0.747). Detailed comparisons are shown in Table 1.

**TABLE 1:** Clinical characteristics of patients in different groups.

	Immunotherapy + chemotherapy (*N* = 22)			Chemotherapy (*N* = 72)			Osimertinib + bevalizumab (*N* = 27)			*F*/*χ* ^2^			*p*		
	1st line osimertinib treatment	2nd/3rd line osimertinib	Total	1st line osimertinib treatment	2nd/3rd line osimertinib	Total	1st line osimertinib treatment	2nd/3rd line osimertinib	Total	1st line osimertinib treatment	2nd/3rd line osimertinib	Total	1st line osimertinib treatment	2nd/3rd‐line osimertinib	Total
Age (years)	61.56 ± 10.30	58.70 ± 10.51	59.86 ± 10.27	61.85 ± 7.63	54.82 ± 11.07	57.46 ± 10.44	62.42 ± 9.22	58.60 ± 9.39	60.30 ± 9.33	0.029	1.109	0.994	0.971	0.336	0.373
Gender															
Male	4	5	9	7	17	24	5	5	10	1.542	0.110	0.456	0.463	0.946	0.796
Female	5	8	13	20	28	48	7	10	17						
Smoking history										3.444	8.396	8.641	0.179	0.015	0.013
Yes	7	7	14	12	20	32	5	1	6						
No	2	6	8	15	25	40	7	14	21						
Pathological type															
Adenocarcinoma	9	13	22	26	45	71	12	14	26				1.000	0.384	0.648
Squamous carcinoma	0	0	0	1	0	1	0	1	1						
Stage															
IIIB–IIIC	0	0	0	3	0	3	1	2	3				0.805	0.070	0.236
IV	9	13	22	24	45	69	11	13	24						
Central nervous metastasis															
Yes	3	1	4	4	8	12	1	2	3	2.467	0.846	0.583	0.291	0.655	0.747
No	6	12	18	23	37	60	11	13	24						
EGFR sensitizing mutation															
19DEL	6	8	14	15	23	38	7	7	14			2.515	0.714	0.782	0.642
L858R	2	5	7	8	16	24	5	6	11						
Other	1	0	1	4	6	10	0	2	2						

### 3.2 Clinical Response

**FIGURE 1:**
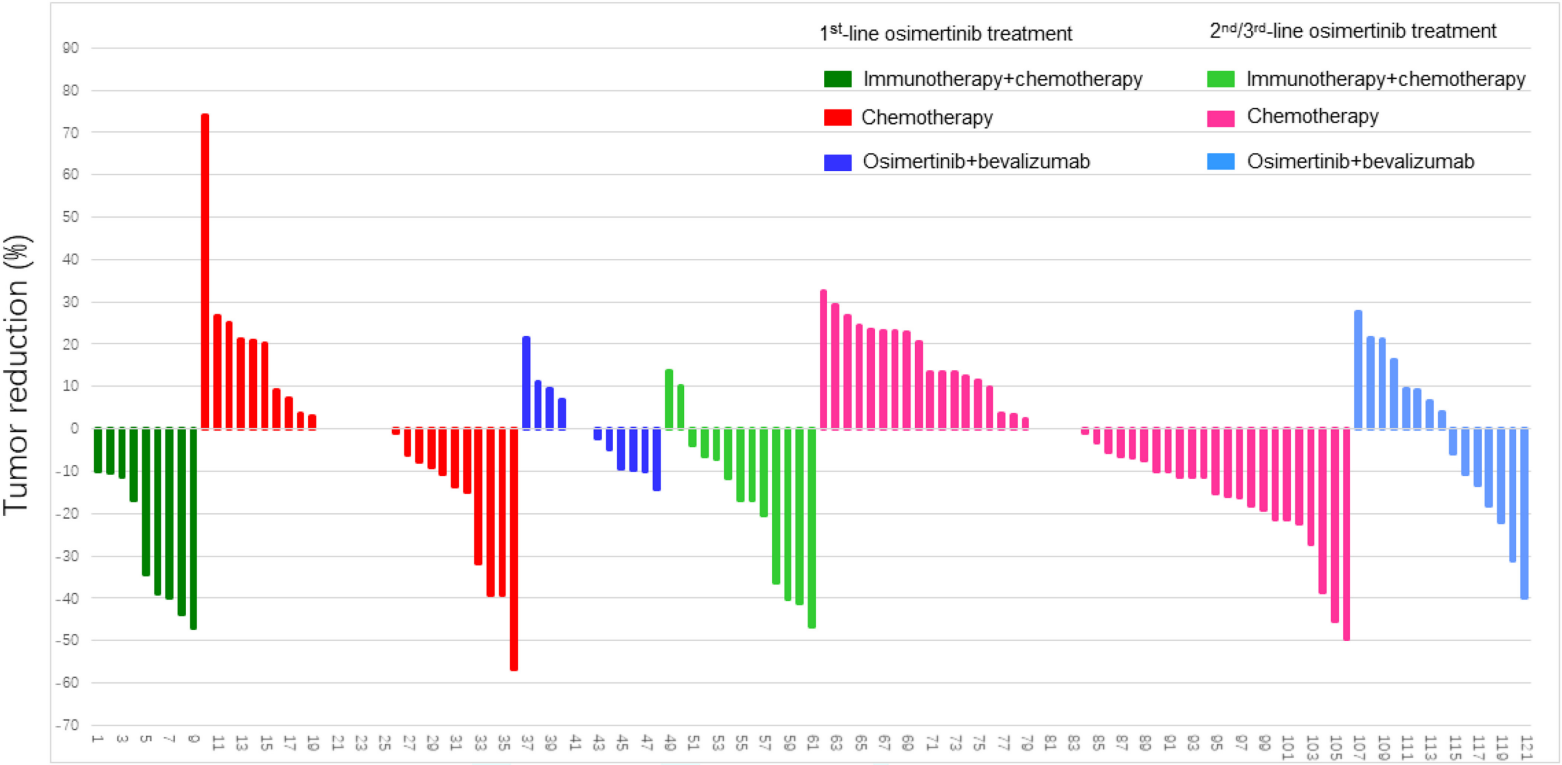
ORRs and DCRs of NSCLC patients with EGFR mutation receiving different treatments after progression on osimertinib.

Clinical response was compared between groups (Figure 1). Our results showed that in patients after progression to 1st line osimertinib therapy, the ORR and DCR were significantly higher in the immunotherapy + chemotherapy group (ORR 55.56%, DCR 100%) than in the chemotherapy group (ORR 14.81%, DCR 77.78%) and osimertinib + bevacizumab group (ORR 0%, DCR 91.67%). For patients following progression on 2nd/3rd line of osimertinib treatment, the ORR and DCR were also significantly higher in the immunotherapy + chemotherapy group (ORR 30.77%, DCR 100%) than in the chemotherapy group (ORR 6.67%, DCR 80.00%) and osimertinib + bevacizumab group (ORR 13.33%, DCR 86.67%).

### 3.3 Comparison of PFS Among Groups

**FIGURE 2:**
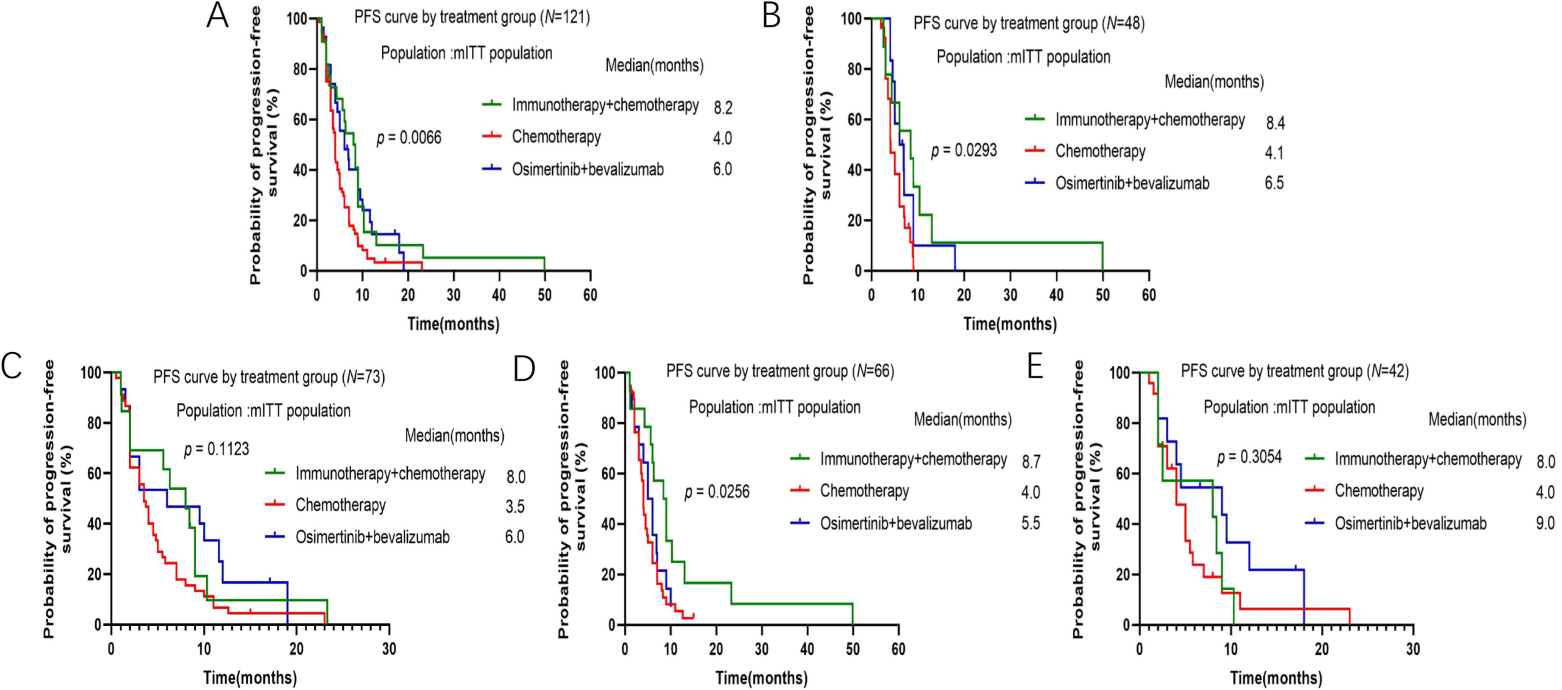
Kaplan–Meier curves of median PFS among patients in different subgroups. (A) All patients; (B) patients after progression on 1st line of osimertinib treatment; (C) patients after progression on 2nd/3rd line of osimertinib treatment; (D) patients with EGFR 19DEL mutation; (E) patients with EGFR 21L858R mutation.

Median PFS (mPFS) in all patients was significantly longer in the immunotherapy + chemotherapy group than in the chemotherapy group and osimertinib + bevacizumab group (mPFS 8.2 months vs. 4.0 months vs. 6.0 months, *p* = 0.0066, Figure 2A). To identify patients most likely to benefit from immunotherapy + chemotherapy, subgroup analysis of mPFS was performed. Patients who progressed on 1st line of osimertinib therapy had significantly longer mPFS in immunotherapy + chemotherapy later line treatment group than the other groups (mPFS 8.4 months vs. 4.1 months vs. 6.5 months, *p* = 0.0293, Figure 2B). Patients after progression on 2nd/3rd line of osimertinib treatment also had numerically prolonged mPFS in immunotherapy + chemotherapy later line treatment group than the other groups, although it did not show significant difference (mPFS 8.0 months vs. 3.5 months vs. 6.0 months, *p* = 0.1123, Figure 2C). Moreover, in patients with EGFR 19DEL mutation, immunotherapy + chemotherapy demonstrated significantly longer mPFS than the other groups (mPFS 8.7 months vs. 4.0 months vs. 5.5 months, *p* = 0.0256, Figure 2D). We then further analyzed mPFS in patients with 19DEL mutation after progression to 1st line or 2nd/3rd line of osimertinib subgroups, respectively. Because the number of patients in each subgroup was small, we only described the mPFS but not to compare between the subgroups (figure did not show). In patients with 19DEL mutation after progression to 1st line of osimertinib therapy, the mPFS in immunotherapy + chemotherapy, chemotherapy group, and osimertinib + bevalizumab group was 9.7 months vs. 4.1 months vs. 6.0 months, respectively. In patients with 19DEL mutation after progression on 2nd/3rd line of osimertinib therapy, the mPFS in these three groups was 8.0 months vs. 3.5 months vs. 6.0 months, respectively. However, in patients with EGFR 21L858R mutation after progression on osimertinib, osimertinib + bevacizumab presented numerically longer mPFS than other therapies although the difference was not significant (mPFS 8.0 months vs. 4.0 months vs. 9.0 months, *p* = 0.3054, Figure 2E). In patients with 21L858R mutation after progression on 1st line of osimertinib treatment subgroups, mPFS of these three groups was 5.5 months vs. 5.0 months vs. 9.0 months, respectively. In patients with 21L858R mutation after progression on 2nd/3rd line of osimertinib therapy, mPFS of these three groups was 8.0 months vs. 4.0 months vs. 6.3 months, respectively.

### 3.4 Comparison of OS Among Groups

**FIGURE 3:**
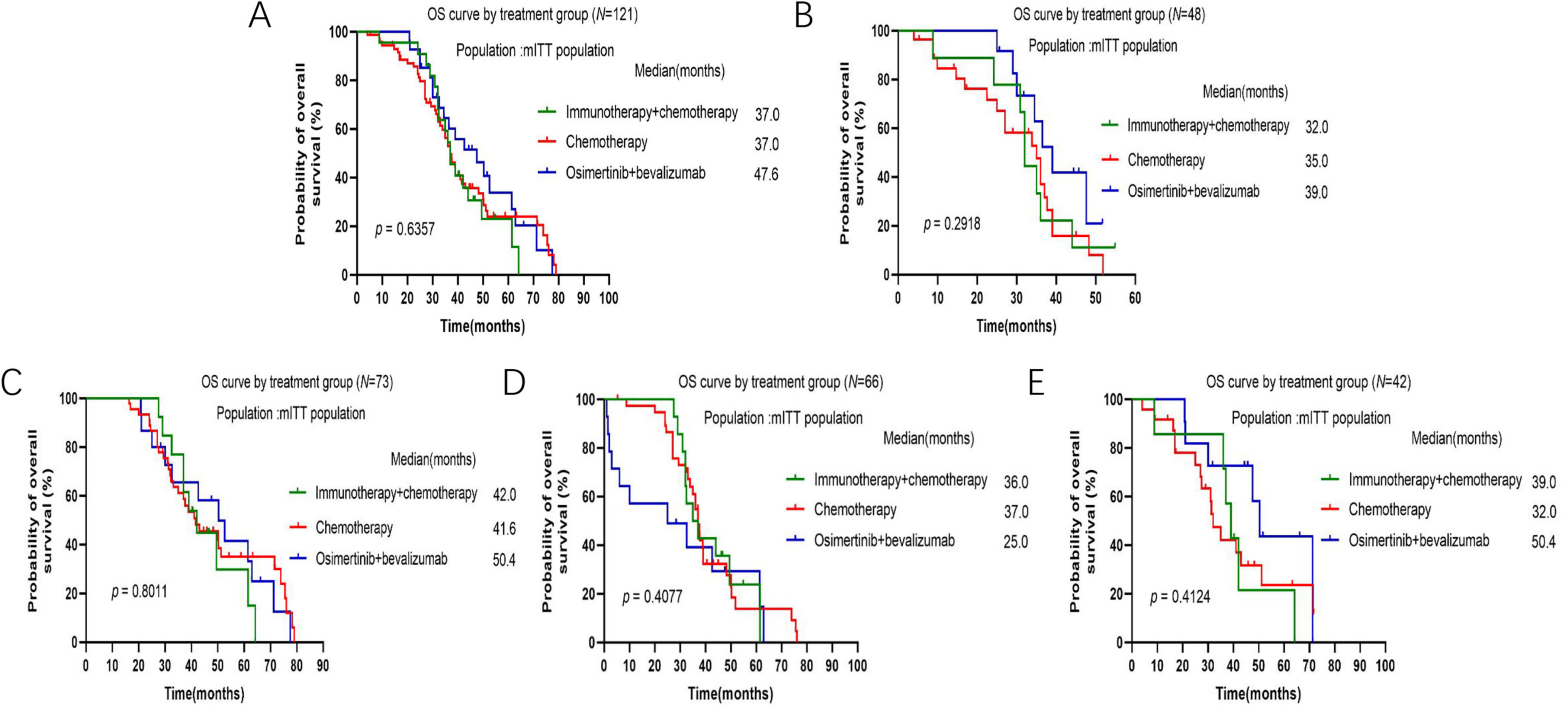
Kaplan–Meier curves of median OS among patients in different subgroups. (A) All patients; (B) patients after progression on 1st line of osimertinib treatment; (C) patients after progression on 2nd/3rd line of osimertinib treatment; (D) patients with EGFR 19DEL mutation; (E) patients with EGFR 21L858R mutation.

The median OS (mOS) of patients after progression on osimertinib did not show remarkable difference among these three groups, although osimertinib + bevacizumab group showed longer mOS numerically (mOS 37.0 months vs. 37.0 months vs. 47.6 months, *p* = 0.6357, Figure 3A). Subgroup analysis was then carried out. In patients after progression on 1st line or 2nd/3rd line of osimertinib treatment, mOS did not show significant difference among these three groups, although osimertinib + bevacizumab group demonstrated longer mOS numerically (Figure 3B,C). For patients with 19DEL mutation after progression on osimertinib, mOS was similar in three groups (mOS 36.0 months vs. 37.0 months vs. 36.5 months, *p* = 0.9191, Figure 3D). For patients with 19DEL mutation after progression to 1st line or 2nd/3rd line of osimertinib treatment, mOS was 33.5 months vs. 37.0 months vs. 34.5 months and 43.3 months vs. 37.0 months and 61.4 months, respectively. Our results also indicated that in patients with 21L858R mutation after progression on osimertinib, osimertinib + bevacizumab group reached a longer mOS than other groups, but the differences did not approach statistical significance (mOS 39.0 months vs. 32.0 months vs. 50.4 months, *p* = 0.4124, Figure 3E). For patients with 21L858R mutation after progression to 1st line or 2nd/3rd line of osimertinib treatment, mOS was 22.4 months vs. 25.0 months vs. 49.7 months and 42.0 months vs. 41.0 months and 40.2 months, respectively.

### 3.5 Comparison of Safety Among Groups

**FIGURE 4:**
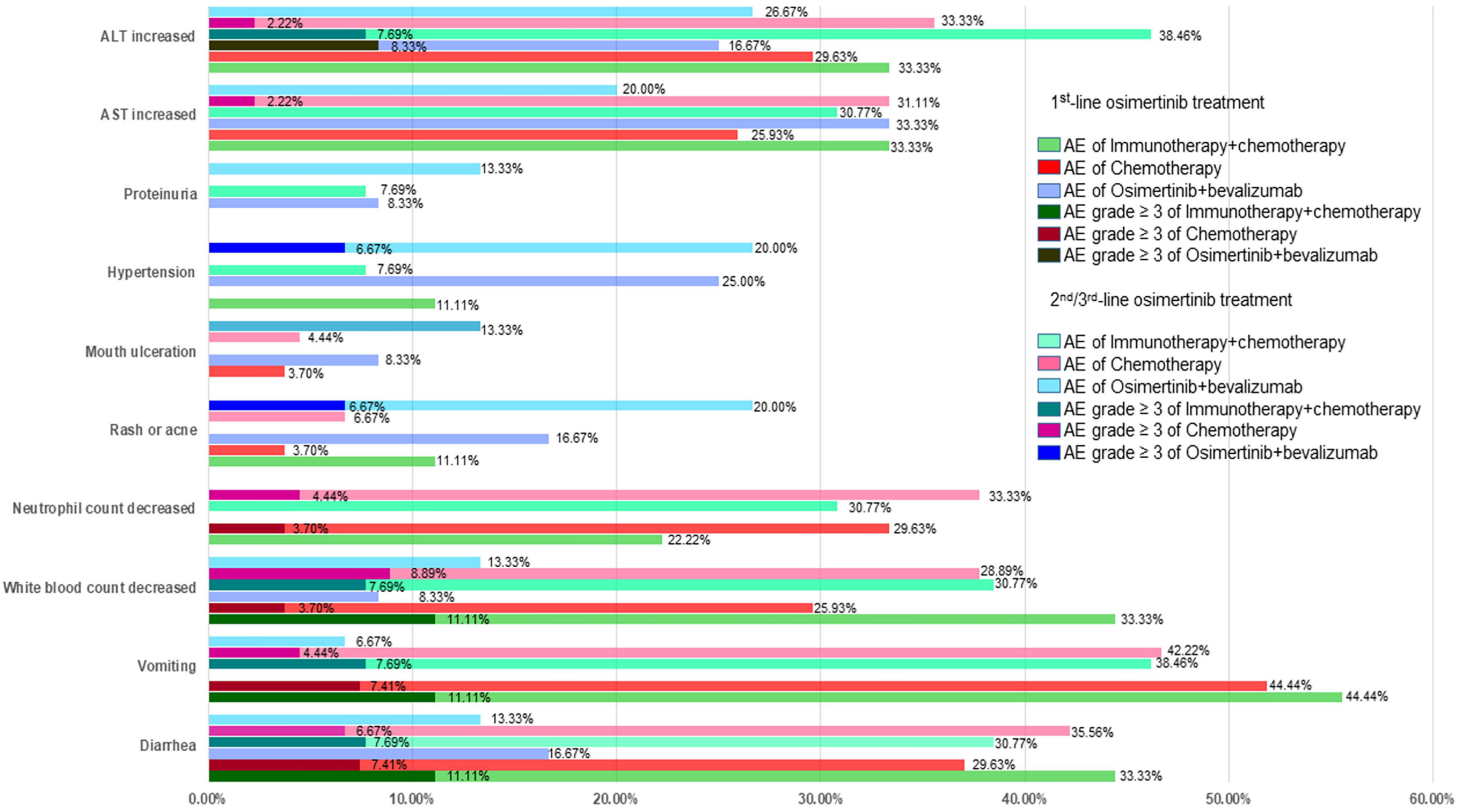
Common treatment‐related adverse events in different groups.

Common TRAEs were recorded and summarized in Figure 4. The most frequent TRAEs in the immunotherapy + chemotherapy group were ALT/AST increased, white blood count decreased, vomiting, and diarrhea (with incidence of 30% or more in patients after progression on both 1st line and 2nd/3rd line of osimertinib subgroups). Similar to patients in the immunotherapy + chemotherapy group, the most frequent TRAEs in the chemotherapy group were neutrophil count decline, white blood count decline, vomiting, and diarrhea (with an incidence of about 30% or more in patients after progression on both 1st line and 2nd/3rd line of osimertinib treatment subgroups). However, in the osimertinib + bevacizumab group, the most frequent AEs were increased ALT/AST, hypertension, rash, or acne (with about 20% or more incidence in patients after progression on both 1st line and 2nd/3rd line of osimertinib treatment subgroups). Patients in the osimertinib + bevacizumab group appeared to have lower neutrophil counts, decreased white blood counts, vomiting, and diarrhea AEs than the other two groups.

## 4 Discussion

The resistance mechanisms of osimertinib were complex, with more than half of the resistance mechanisms still unknown. Chemotherapy is considered as the standard treatment for patients after progression on osimertinib with unknown resistance mechanisms. In our study, we demonstrated that NSCLC patients with unknown resistance mechanism after osimertinib progression treated with immunotherapy + chemotherapy achieved better ORR and PFS than those treated with chemotherapy or osimertinib + bevacizumab. While OS was similar in three groups with numerically longer OS of osimertinib + bevacizumab in some subgroups. AEs were less observed in osimertinib + bevacizumab than other groups. In conclusion, our study suggested that NSCLC patients, after progression on osimertinib with unknown resistance mechanisms, may benefit from immunotherapy + chemotherapy and osimertinib + bevacizumab.

A series of studies such as CheckMate153 and IMPOWER130 have shown that NSCLC patients with EGFR or ALK mutations hardly benefit from immunotherapy alone [20, 24, 25, 26]. However, whether NSCLC patients with EGFR mutation after prior EGFR‐TKI treatment may benefit from combined immunotherapy is still under discussion. IMPOWER 150 study revealed that combination of Atezolizumab with chemotherapy and bevacizumab treatment improved survival in patients with EGFR mutation after progression on TKIs when compared with chemotherapy plus bevacizumab therapy [21]. Zhang's study also indicated that anti‐PD‐1 inhibitors toripalimab plus chemotherapy showed a promising anti‐tumor efficacy with a tolerable safety profile for NSCLC patients after progression on EGFR TKI therapies (ORR 54.8%, mPFS 7.6 months) [27]. However, CheckMate 722 study showed opposite results that Nivolumab combined with chemotherapy did not achieve prolonged survival than chemotherapy in patients with EGFR mutation after progression on 1st/2nd line of EGFR‐TKI treatment (mPFS 5.6 months vs. 5.4 months, *p* = 0.0528; mOS 19.4 months vs. 15.9 months, HR = 0.82, 95% CI 0.61–1.10) [28]. Meanwhile, White reported that the combination of immunotherapy and chemotherapy was associated with a worse OS compared with chemotherapy alone in patients after progression on osimertinib [29]. Whereas our present study suggested that immunotherapy plus chemotherapy achieved higher ORR and prolonged PFS in patients with unknown resistance mechanisms following osimertinib progression compared with chemotherapy alone or osimertinib + bevacizumab therapy, especially in patients with EGFR 19DEL mutation. Patients after progression on both 1st line or 2nd/3rd line osimertinib therapy may all benefit from immunotherapy plus chemotherapy, especially for those after progression on 1st line osimertinib therapy. In the study ABC‐lung, atezolizumab plus bevacizumab and chemotherapy (pemetrexed or carboplatin/paclitaxel) showed benefit in NSCLC patients progressed on EGFR‐TKIs (ORR: 32%–47%, mPFS: 6.4–7.6 months) [30]. In our study, the ORR and mPFS of immunotherapy + chemotherapy group were 55.56% and 8.2 months, which seemed higher to the data of ABC‐lung [30]. We deduced inconsistencies in different inclusion criteria and anti‐PD‐1/PD‐L1 inhibitors used in different studies. Kohsuke's research discovered that after treatment of EGFR‐TKI, the expression of PD‐L1 and TMB levels of patients were upregulated. EGFR‐TKI therapy has been associated with changes in the tumor immune microenvironment of EGFR‐mutated NSCLC, which may provide clues for further optimization of PD‐1 inhibitor therapy [31].

In our present study, we noticed that although mOS did not show significant difference among these three groups, mOS of osimertinib + bevacizumab was numerically longer in some subgroups, especially in patients with EGFR 21L858R mutation subgroup. Previous research has shown that for patients with progressive progression after EGFR‐TKI treatment, early combination therapy may provide benefits over EGFR‐TKI alone [32, 33, 34]. A series of clinical trials provided evidence that the first‐line combination of EGFR‐TKI and bevacizumab showed significant prolonged PFS in NSCLC patients [35, 36]. Long's study revealed that continuous original TKI combined with bevacizumab showed partly favorable efficacy with 8 months of PFS2 and safety in EGFR‐mutant NSCLC patients experiencing gradual progression after EGFR‐TKI treatment [23]. Cui's study also suggested that osimertinib + bevacizumab prolonged PFS (7.0 months vs. 4.9 months, *p* = 0.001) and OS (12.6 months vs. 7.1 months, *p* = 0.001) than chemotherapy + bevacizumab in NSCLC patients after progression on osimertinib [37]. In consistent with these studies, our present study also noted that the mPFS of the osimertinib + bevacizumab group was longer than that of the chemotherapy group, but shorter than that of the immunotherapy + chemotherapy group. As for OS, although OS was similar in three groups, the osimertinib + bevacizumab group appeared to have numerically longer mOS in NSCLC patients with unknown resistance mechanisms after osimertinib progression. The OS was influenced by a number of factors, including each line of treatment received of patients. The reason is unclear. We speculate that patients treated with osimertinib + bevacizumab after osimertinib progression may receive immunotherapy + chemotherapy or chemotherapy in the later lines, but most patients treated with immunotherapy + chemotherapy or chemotherapy after osimertinib progression may not receive osimertinib + bevacizumab in the later lines of treatment. Different lines of treatment may affect the OS of patients in different groups.

In terms of safety, compared with immunotherapy + chemotherapy and chemotherapy, osimertinib + bevacizumab TRAEs were milder, especially in AEs related to gastrointestinal and bone marrow suppression. Osimertinib + bevacizumab had its own set of TRAEs such as hypertension, proteinuria, rash, or acne, which were observed less in the other two groups. Most TRAEs were manageable and no treatment‐related deaths were observed in our study, suggesting that immunotherapy + chemotherapy and osimertinib + bevacizumab were both well tolerated in NSCLC patients after osimertinib progression.

Some limitations of our studies should be noted. First, a retrospective study with a relatively small sample size may inevitably introduce collection bias and weaken the level of evidence. Second, the patients in our study received different immunotherapy and chemotherapy regimens. Disuniformity in treatment may affect results. Third, PD‐L1 expression and co‐mutation status were not accessed, making it difficult to analyze what kind of patients would benefit from immunotherapy + chemotherapy or osimertinib + bevacizumab treatment after osimertinib progression. Therefore, perspective studies with larger sample sizes are needed to verify our findings.

In conclusion, our study provides clinical evidence that NSCLC patients after progression on osimertinib with unknown resistance mechanisms may benefit from immunotherapy + chemotherapy, with higher ORR and longer PFS compared with osimertinib + bevacizumab or chemotherapy groups. Osimertinib + bevacizumab treatment was also an optional option for patients after osimertinib progression, as OS was numerically longer in this group, particularly in patients with EGFR 21L858R mutation and also safer.

## Author Contributions

Xin Liao and Tingting He wrote the main manuscript text. Pian Liu and Jing Li were responsible for patient data collection and data collation. Xiong Wan completed statistical analysis. Yubo Wang and Yong He were responsible for the final review of the manuscript text.

## Ethics Statement

This study was supervised and approved by the Ethics Committee of Chongqing University Jiangjin Hospital.

## Conflicts of Interest

The authors declare no conflicts of interest.

## Data Availability

Research data are not shared.
